# Spatial Heterogeneity and Risk Maps of Community Infestation by *Triatoma infestans* in Rural Northwestern Argentina

**DOI:** 10.1371/journal.pntd.0001788

**Published:** 2012-08-14

**Authors:** Gonzalo M. Vazquez-Prokopec, Cynthia Spillmann, Mario Zaidenberg, Ricardo E. Gürtler, Uriel Kitron

**Affiliations:** 1 Laboratorio de Eco-Epidemiología, Universidad de Buenos Aires, Buenos Aires, Argentina; 2 Department of Environmental Studies, Emory University, Atlanta, Georgia, United States of America; 3 Fogarty International Center, National Institutes of Health, Bethesda, Maryland, United States of America; 4 Coordinación Nacional de Control de Vectores, Ministerio de Salud de la Nación, Córdoba, Argentina; Mahidol University, Thailand

## Abstract

**Background:**

Fifty years of residual insecticide spraying to control *Triatoma infestans* in the Gran Chaco region of northern Argentina, Paraguay and Bolivia shows that vertically coordinated interventions aiming at full coverage have limited effects and are unsustainable. We quantified the spatial distribution of *T. infestans* domestic infestation at the district level, identified environmental factors associated with high infestation and then explored the usefulness of risk maps for the spatial stratification of interventions.

**Methods and Findings:**

We performed spatial analyses of house infestation data collected by the National Chagas Service in Moreno Department, northern Argentina (1999–2002). Clusters of high domestic infestation occurred in the southwestern extreme of the district. A multi-model selection approach showed that domestic infestation clustered in areas of low elevation, with few farmlands, high density of rural houses, high mean maximum land surface temperature, large NDVI, and high percentage of degraded and deforested lands. The best model classified 98.4% of the communities in the training dataset (sensitivity, 93.3%; specificity, 95.4%). The risk map evidenced that the high-risk area only encompassed 16% of the district. By building a network-based transportation model we assessed the operational costs of spatially contiguous and spatially targeted interventions. Targeting clusters of high infestation would have reached ∼80% of all communities slated for full-coverage insecticide spraying, reducing in half the total time and economic cost incurred by a spatially contiguous strategy.

**Conclusions and Significance:**

In disperse rural areas where control programs can accomplish limited coverage, consideration of infestation hot spots can contribute to the design and execution of cost-effective interventions against Chagas disease vectors. If field validated, targeted vertical control in high risk areas and horizontal control in medium to low risk areas may provide both a logistically and economically feasible alternative to blanket vertical insecticide spraying when resources are limited.

## Author Summary

In the Southern Cone countries of South America, vectorborne transmission of Chagas disease persists in the Gran Chaco region of northern Argentina, Paraguay and Bolivia, where *Triatoma infestans* is the main vector. More than 50 years of vector control in this region demonstrate that vertically coordinated spraying campaigns aiming at full coverage are unsustainable in practice. Understaffed programmes are challenged by the number of households and vast extension of the endemic area, resulting in suboptimal insecticide coverage and limited and shortlived impacts on vector populations. Our rationale was to recognize this inherent limitation and provide a scientific base for the improvement of current interventions. We quantified the spatial distribution of *T. infestans* at the Department level and found that environmental and demographic factors can be used to predict the occurrence of areas with high risk of vector infestation that can then be targeted for control. In rural and dispersed areas such as the Gran Chaco, mapping surrogates of *T. cruzi* transmission risk and incorporating spatial heterogeneities in vector distribution into control and surveillance strategies can contribute to improved delivery of interventions and to the sustainable control and management of Chagas disease.

## Introduction

Over the past 15 years, the burden of Chagas disease has been significantly reduced (from an estimated ∼30 million human cases in 1990 to ∼9–11 million in 2006) as a consequence of direct actions promoted by several multinational regional initiatives [1], [2]. The key for such success has been the long-term implementation of residual insecticide applications to kill domestic triatomine bugs, the screening of blood donors for the presence of the flagellate parasite *Trypanosoma cruzi*, and the treatment of infected infants born to infected mothers [2]. In the Southern Cone of South America, *T. cruzi* transmission by the main vector, *Triatoma infestans*, was interrupted in Uruguay, Chile, Brazil and parts of Argentina and Paraguay [1], [2]. However, only limited success was attained in the Gran Chaco region of northern Argentina, Bolivia and Paraguay (the core of *T. infestans* distribution) where Chagas disease is still highly prevalent [3].

Vector control activities in this impoverished and mostly rural area are severely hampered by economic, logistic and political constraints, resulting in sporadic spraying of households with pyrethroid insecticides, with the consequent high bug reinfestation rates of rural communities [3]–[5]. Furthermore, the lack of effectiveness of pyrethroid insecticides in peridomestic habitats [6]–[8] coupled with the recent finding of *T. infestans* sylvatic populations [9]–[11] and the emergence of pyrethroid resistance in multiple localities [12]–[15] render the elimination of *T. infestans* from the Gran Chaco an elusive challenge. New approaches, tools, and methods for Chagas disease vector control and disease prevention are urgently needed.

The distribution of *T. infestans* infestations and *T. cruzi* transmission are highly heterogeneous, with a few premises, households or communities accounting for a significant fraction of bugs and/or parasite transmission [16]–[20]. Knowledge of the location of households or communities that are at highest risk can be used to target vector control interventions for maximum effect (i.e., spatial targeting) in comparison to blanket or untargeted interventions that may fail to reach some of the high-risk households or communities in an effective manner [21], [22]. Spatially targeted interventions tailored for local contexts of parasite transmission risk, vector biology and insecticide resistance can constitute the backbone of an integrated Chagas disease vector management strategy in the Gran Chaco.

Most knowledge on the factors affecting the spatial distribution of *T. infestans* has emerged from village-wide surveys performed at the premise/household level [4], [19], [23]–[28]. Given the large amounts of data and detail needed for mapping transmission risk, this fine spatial scale is not appropriate for planning and implementing large-scale vector control interventions. Rather, district-wide (e.g., state or county equivalents) entomologic and epidemiologic information collected at the community-level may represent the best compromise between the level of empirical detail needed and potential applicability for the design of vector control interventions. Unfortunately, to date few studies have quantified the spatial distribution of *T. infestans* and the factors associated with the distribution of bug infestation at the district level [7], [18], and none have developed risk maps of *T. infestans* distribution and *T. cruzi* transmission at this scale.

Extending from previous research on the cost-effectiveness of Chagas disease vector control interventions [5], and as part of a larger project on the eco-epidemiology and control of Chagas disease in the Argentinean Chaco, the present study aimed to: a) quantify the spatial distribution of domestic infestation by *T. infestans* at the district level in the absence of recent vector control actions; b) identify environmental factors responsible for the heterogeneous occurrence of bug-infested communities; c) develop risk maps of domestic infestation and, based on such information, d) evaluate the relative costs of reaching and spraying communities predicted to be at high risk of domestic infestation by *T. infestans*.

## Materials and Methods

### Study area

The Moreno Department (centroid at 62°26′W, 27°15′S), located in the Province of Santiago del Estero, northwestern Argentina, covers an area of 16,788 km^2^ (Figure S1). Moreno is located in the dry Chaco region, a semiarid plain with hardwood forest under intensive exploitation characterized by an average total rainfall of 549 mm and a rainy season from October to May [29]. In 2001 Moreno had approximately 25,000 habitants and 5,439 houses of which 2,911 (54%) were rural houses located in 275 communities [30]; most (75%) of the rural communities consisted of 1–10 houses (Figure S1). Rural houses usually have adobe walls and thatched roofs, one or two bedrooms, and a 5–10 m wide veranda in the front. The peridomestic environment includes structures that do not share a roof with the bedrooms, such as storerooms, chicken coops and corrals. In 2001, almost 37% of all houses in the department had unmet basic needs (an index that combines lack of adequate housing, tap water, crowding, and income) [30]. Exploitation of forest resources (hardwood for charcoal and logs, hunting), raising goats (and cattle), and subsistence agriculture are the main sources of income of rural villagers. Historically, soybean and cotton have been cultivated in eastern Moreno but recent increases in rainfall had favored the introduction of genetically modified soybean, expanding the agricultural frontier towards the center of the Department.

### Data sources and management

A complete database with information on the number of domiciles per community infested by *T. infestans* was generated from records provided by the National Chagas Service (NCS, Argentine Ministry of Health) in the year 2006 [5]. Bug infestation data originated from householders' notifications to community leaders and vector control personnel as part of the horizontal control program implemented in Argentina since 1993 [5]. Data included only anonimized records and was provided by NCS via a data sharing agreement with the University of Buenos Aires. The prevalence of infestation (i.e., infestation index) in 25 communities during 1999–2001 was positively correlated with the infestation prevalence assessed by timed manual collections performed by NCS staff in the same communities in 2002 in domiciles (Pearson correlation, r = 0.45, *P* = 0.02), but not in peridomiciles (r = 20.14, *P* = 0.4). Because householders' reports likely underestimated peridomestic infestation (with which they have much less contact [31]), such information will not be analyzed in this study. We also excluded from all our analyses the three main cities of Moreno (totaling ∼2,528 houses) because vector-borne transmission of *T. cruzi* in this area is considered to be mostly rural. Only information for the years 1999–2002 (the period for which almost all Moreno communities had infestation data) was used, since we aimed to reach maximum spatial coverage of all rural communities in the Department within a short time-frame. After the year 2002, vector control and surveillance activities in Moreno have been scarce, making of the analyzed dataset the best and most current in terms of spatial coverage and quality of entomologic data. Vector control activities in the Department from 1997 to 1999 were negligible because of lack of insecticides and NCS personnel [5].

A base digital map of the Moreno Department including the location of rural villages, main waterways and road infrastructure at a 1∶250,000 scale was obtained from Instituto Geográfico Militar of Argentina. A georeferenced Landsat 7 ETM+ (NASA; http://landsat.gsfc.nasa.gov/) satellite image (spatial resolution 28.5×28.5 m) from October 2002 and cadastral paper maps from Santiago del Estero Province were used to digitize communities that were not present in the base digital map using ArcGIS 10 (ESRI, Redlands, CA). The final map was then projected in Universal Transverse Mercator (UTM), Zone 20S, WGS1984 datum, and the distance matrix from all the digitized communities associated with the NCS entomologic database.

Several data sources were a priori selected to calculate environmental parameters deemed important in explaining the spatial distribution of *T. infestans* in Moreno. The scale of analysis (i.e., individual communities) prevented the use of census-derived socio-demographic data, which are available at a much coarser scale (Moreno is divided in 10 census units aggregating data from rural communities and urban centers). Instead, we considered environmental variables describing different attributes of the landscape rural populations depend on for subsistence, such as land-use type, elevation and NDVI. Additionally, other environmental factors such as temperature (impacting *T. infestans* population dynamics and pyrethroid insecticide effectiveness) and density of rural houses (delineating areas of high population density) were considered.

The raw pixel values of the Landsat ETM+ image were first converted to surface reflectance [32] and then classified using an unsupervised method into five classes describing the degree of landscape modification in Moreno: bare soil; croplands; deforested lands for cattle raising (a savanna-like landscape with grasses and very few trees); degraded forest (natural forest in which tall trees were extracted and only scrubs prevail), and undisturbed forest (Figure S1). The overall accuracy of data classification (kappa statistic = 0.82) was assessed from 46 ground control points digitized from a high resolution Ikonos image (Space Imaging, Atlanta, GA) from October 2002 using Erdas Imagine 9.0 (Erdas, Norcross, GA). The Landsat ETM+ image was later used to estimate the Normalized Difference Vegetation Index (NDVI) by calculating the spectral difference between the red and infrared bands. Elevation (in meters) of all communities was derived from the Shuttle Radar Topography Mission (SRTM; http://seamless.usgs.gov) Digital Terrain Elevation data (∼90 m horizontal resolution and 1 m vertical resolution). Land surface temperature (LST) for the year 2002 was derived from Moderate Resolution Imaging Spectroradiometer (MODIS; http://modis.gsfc.nasa.gov/) images (∼1 km spatial resolution and 1°C accuracy). A total of 36 images with 8-day LST averages were used to calculate the mean maximum LST (in degree Celsius) throughout the Moreno Department during the year 2002. The remaining 10 images (making up the 46-image dataset for 2002) were excluded from the analysis due to their high (>20%) cloud cover. NDVI and mean maximum LST were two of the most important climatic variables explaining the spatial distribution of *T. infestans* at the continental and district-wide levels [7], [19].

A 2 km circular buffer area was created around the centroid of each rural community (in Moreno, most rural houses are found within 2 km of the community center) to characterize its environmental attributes by calculating the percentage of pixels belonging to each land-use class and the average NDVI, LST and elevation using ArcGIS 10.

### Statistical analysis

A local spatial statistic (Getis Gi*(d), [33], [34]) was applied to identify the precise location of clusters or “hot spots” of high prevalence of domestic infestation by *T. infestans*. The area nearby the centroid of each rural community was searched at increasing distances (*d*) for occurrence of communities with higher prevalence of infestation values than expected by 999 Monte Carlo randomizations [33], [34]. Communities were identified as members of positive or negative clusters when the Gi*(d) value at distance *d* was higher or lower than the Monte Carlo expectation, respectively. Significance was assessed at an alpha of 0.05. To identify the distance up to which clustering was maximized, we plotted the absolute value of the sum of Gi*(d) (Σ|Gi*(d)|) over 1 km increments and identified the distance at which such value was the highest. We performed an edge effect correction of the Gi*(d) statistic by including prevalence of infestation values from communities located in neighboring Departments (Figueroa, Ibarra and Alberdi) up to a distance of 25 km from the Moreno border (buffer area edge effect correction, [33]). For all analyses, the distance radius up to which clustering was evaluated was 40 km (one-third of the shortest dimension of the department). We consider the Gi*(d) test to be more suitable for our analysis in comparison to Kulldorff Spatial Scan test because the latter tends to overestimate the contribution of isolated households/small communities to the overall pattern of *T. infestans* distribution (due to the estimation of high log-likelihood values, indicative of clustering, encompassing isolated infested households).

A random selection of 80% of the 220 communities with full entomologic, human demographic and environmental data was used as a training dataset to identify the factors associated with the prevalence of domestic infestation (multiple linear regression) or the membership in a cluster of high *T. infestans* domestic infestation (multiple logistic regression). Inference was based on a multi-model selection approach [35]. Under this analytic framework a set of candidate models are contrasted with each other and the best model (or small set of good models) selected given the support received from the data [35]. We evaluated model fit using the Akaike information criterion (AIC) where the best model had the lowest AIC value and differed from the next best model by at least 2 units [35]. Models with AIC values within 2 units were considered equally good in predicting the data [35]. We further estimated the Akaike weight (ω_i_) for each model, describing the probability that a particular model was the best given the candidate set of models [35]. For each independent factor (*j*) evaluated we estimated its sum of Akaike weights (Σω_i_ *(j)*) as the sum of the ω_i_ of the models in which variable *j* was included [35]. This metric (bounded between 0 and 1) allows determination of the relative importance of each independent variable in predicting the data [35].

Model fit was evaluated by comparing the model predictions with data from the remaining 20% communities (test dataset). Since the predicted values of the logistic regression were represented by probabilities, we used the Youden index [36] to identify the optimal probability cut-off point to classify a community as either member or non-member of a cluster of high *T. infestans* domestic infestation. Briefly, the Youden index is the probability value at which the sum of sensitivity and specificity are maximum [36]. The product of the regression coefficients from the best model and their respective rasterized GIS layers was then integrated into the logistic function to generate a predictive map showing the probability of membership to a high *T. infestans* domestic infestation cluster. The input GIS layers and the scale of the risk map were set to a spatial scale of 1 km^2^ (the coarser scale of the input data, belonging to the MODIS LST estimates).

We used the results from the developed risk maps to explore the utility of novel analytical approaches for the design of spatially targeted interventions. Our premise was that risk maps could be used as predictive tools to identify the areas of high bug infestation where interventions could be targeted. First, we developed a scenario under which a blanket spraying campaign reached every community (i.e., following the rule of contiguity in which brigades visit the nearest neighbor of a treated community). In a second scenario, we used the developed risk map to guide the selection of areas of predicted high infestation clustering where interventions were targeted (i.e., a spatially targeted approach). We used the Vehicle Routing Problem (VRP) solver within the Network Analyst extension of the ArcGIS 10.0 software (ESRI, Redlands, CA) to calculate the total distance and time it will take two teams of two technicians each to reach those communities slated for full insecticide spraying (i.e., with domestic infestations higher than 10%) under each scenario.

The VRP algorithm finds the best (i.e., lowest cost) route for a fleet of vehicles moving over a road network by implementing a multidimensional version of the Traveling Salesman Problem (TSP). Given a list of communities and their pair-wise distances, a TSP algorithm tries to find a shortest possible tour that visits each community exactly once [37]. The VRP is a modification of the TSP algorithm because it uses the pair-wise matrix to assign community-to-depot trips, one at a time, to the most appropriate route (the algorithm requires 2 or more vehicles to generate the routes). The initial solution is then improved upon by re-sequencing the trips on each route, as well as by providing mobility constraints such as priority areas or adding/removing vehicles. In the VRP, therefore, depots (the home base of the spraying teams) and communities can be visited more than once to optimize the spatial allocation of vehicles. Levy et al. [21] had recently applied the TSP algorithm to identify the ordering of districts within a city for the rational spraying of insecticides against *T. infestans*. Our analysis expands on such work by applying the VRP algorithm, by considering multiple vehicles and the explicit road network (rather than districts), and by comparing two spatially-explicit vector-control strategies suitable for rural communities.

Our model was spatially and temporally explicit, and had the following attributes: a) due to resource constraints, only two trucks with two technicians each were available for spraying; b) each truck was stationed in one of the two main cities of Moreno (Quimili, Moreno's capital and Tintina, the district's second largest city); c) each team worked 8 hours a day and spent an average of 2 hours spraying a single rural household (such time accounted for spraying and breaks); d) calculations considered travel time from each city to every community and from community to community; e) if a community had more houses than the ones a team could spray on a single day, the model accounted for the overnight stay of the team in the village until spraying of the community was completed; e) each team was scheduled to spray half the communities in the Department; f) a community can only be sprayed once (by the team that reaches it the earliest); g) mobility using paved roads was faster (and preferred) than on dirt roads; and h) for the spatially-targeted strategy, communities with the highest risk of *T. infestans* infestation were prioritized for control at the beginning of the campaign. The model outputs included a per-community summary of the distance traveled to reach it and the total time spent spraying it, and a cumulative summary of the total distance covered and the duration of the spraying campaign. The model did not account for days lost due to climatic constraints (rainfall or windy conditions, not favorable for insecticide spraying) or vehicle malfunction. We considered the unit costs (in US$ of 2009) of salaries, per-diem, insecticides and gasoline calculated for a vertical strategy by Vazquez-Prokopec et al. [5] for the Moreno Department to estimate the overall cost of each modeled scenario.

Spatial analyses were performed using PPA (Chen and Getis 1998, San Diego State University, San Diego, CA) and ClusterSeer (TerraSeer, Ann Arbor MI) software, whereas non-spatial analyses were performed with STATA 9.1 (Stata Corp).

## Results

### Spatial pattern of infestation

**Figure 1:**
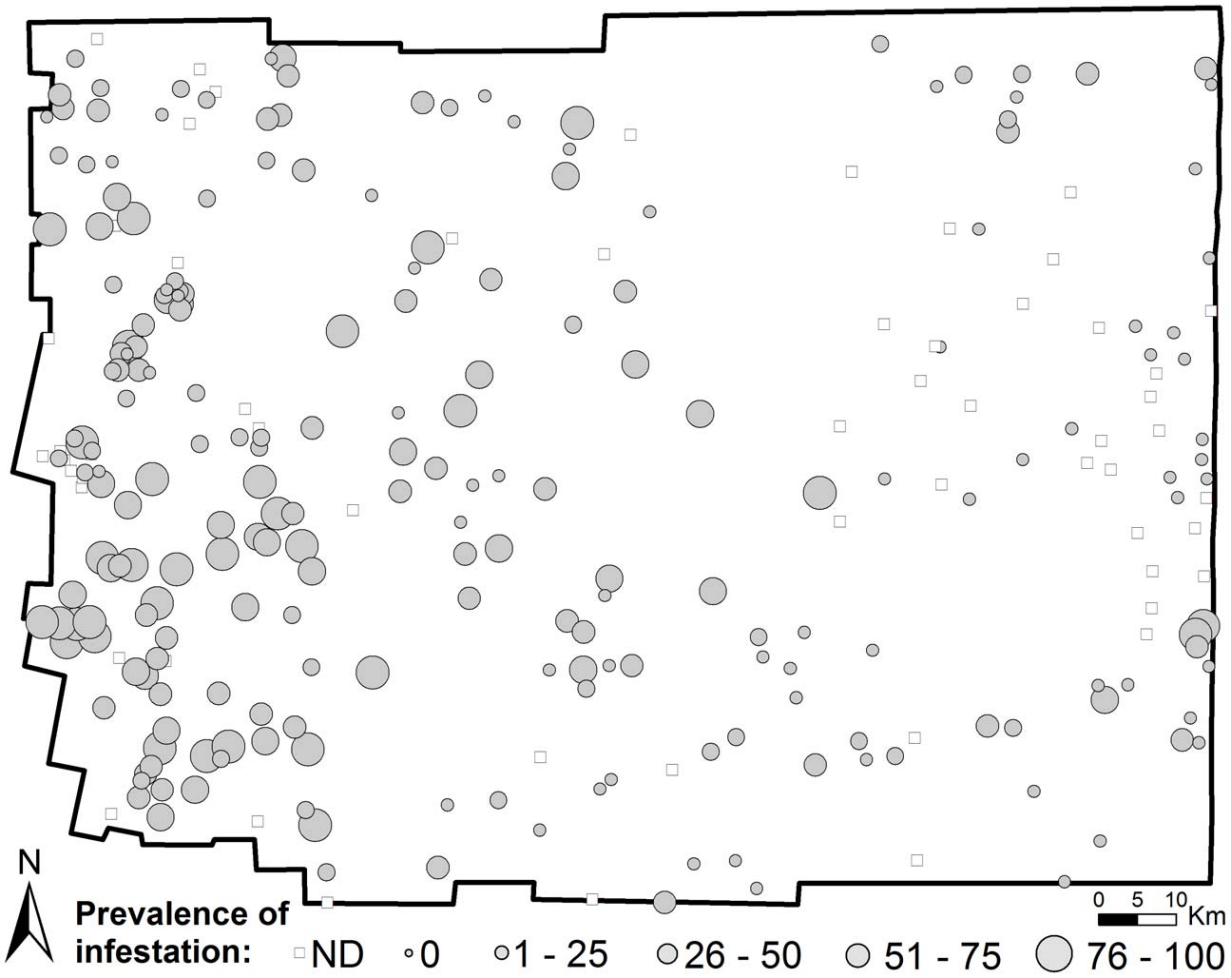
Spatial distribution of *T. infestans* domestic infestation. Prevalence of domestic infestation by *T. infestans* (assessed by householders' collections) during 1999–2002 in the Moreno Department, Santiago del Estero, Argentina. ND refers to communities for which infestation data were not available.

A total of 220 rural communities (80% of the total 275 rural communities) presented entomological data during 1999–2002 (Figure 1). Most (93%) communities without entomological data were small rural settlements with 1–4 houses. Overall, 29.7% of houses (857 of 2,885 houses) were found infested with *T. infestans*. The average prevalence of infestation across communities was 34.9% (SD = 33.5%). A total of 63 communities (28.6%) were negative for domestic infestation, whereas 81 communities (36.8%) reported domestic prevalence values of 50% or more. The average distance (± SD) from the centroid of an infested community to the nearest infested community was 4.3±3.4 km (Figure 1). Communities located in the southwestern quadrant of the Department presented a significantly higher prevalence of domestic infestation by *T. infestans* than communities located anywhere in the remaining quadrants (Mann-Whitney, U = 2.45; d.f. = 1; *P*<0.001) (Figure 1).

**Figure 2:**
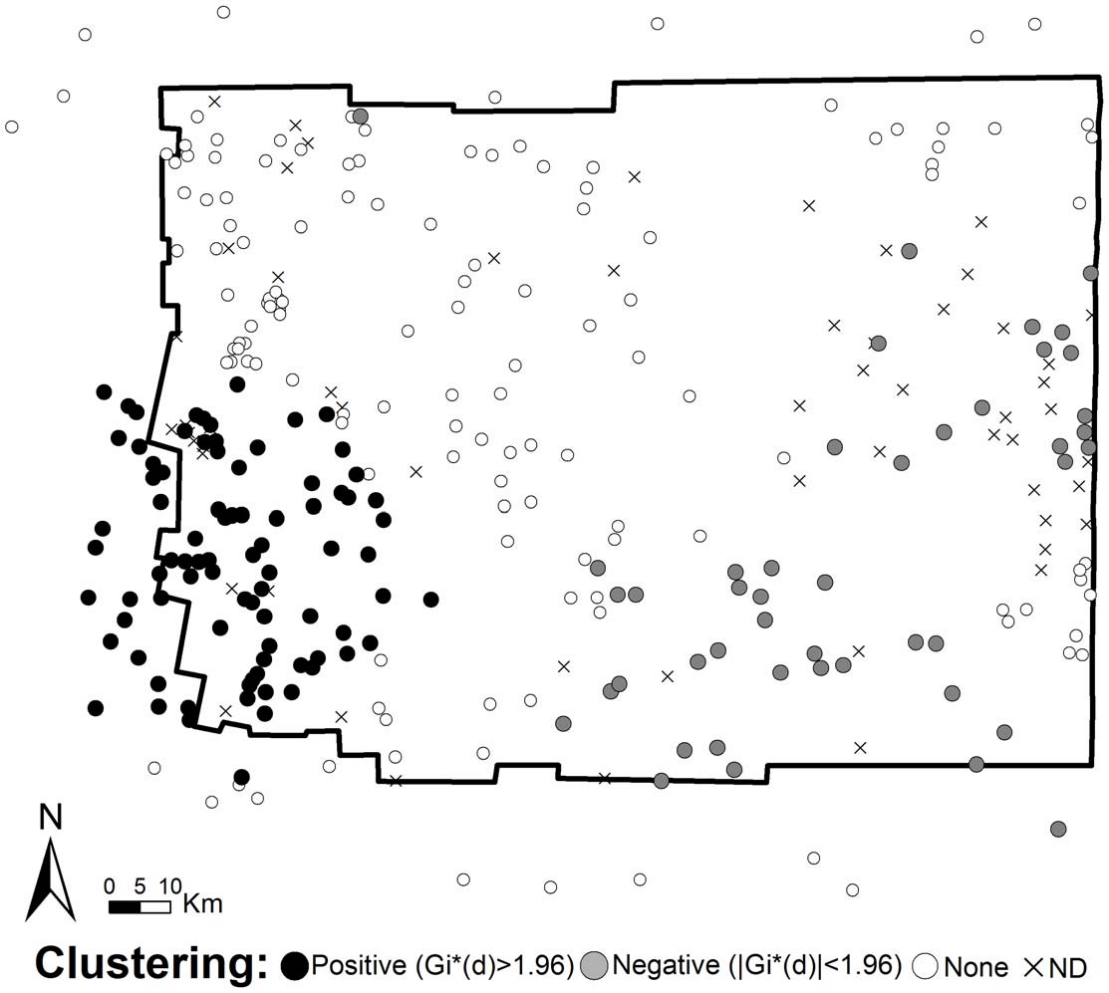
Local clustering of *T. infestans* domestic infestation. Location of significant clusters of domestic infestation in the Moreno Department, Santiago del Estero, Argentina. Positive clusters indentify communities with significantly high prevalence values, whereas negative clusters identify communities with significantly low prevalence values.

Clustering of domestic infestation (quantified as the absolute sum of G_i_*(d)) was maximized at a distance of 24 km (Figure S2). At this distance range, a total of 60 (27.3%) communities belonged to a unique cluster of high domestic infestation, whereas 45 (20.5%) communities belonged to a cluster of low domestic infestation (Figure 2). The cluster of high domestic infestation occurred in the southwestern extreme of Moreno, whereas the clusters of low domestic infestation in the center and east of the Department (Figure 2). Clustering of high domestic infestation extended over the neighboring Figueroa Department, proving that the edge effect correction was effective in accounting for spatial variation along the edges. The prevalence of domestic infestation inside the area of positive clustering (median, Q1–Q3; 41%, 33–65%) was two times higher than outside the clustering area (21%, 0–50%) (U = 1.346; d.f. = 1; *P*<0.001).

### Factors explaining spatial heterogeneity

We evaluated the effects of various a-priori selected environmental factors on the prevalence of domestic infestation and the membership of a community in a cluster of high domestic infestation by *T. infestans*. Table 1 shows the results of the multi-model selection approach, ranking the top 10 linear regression models (of 20 models evaluated) from best to worst. The Akaike weight (the probability that model *i* is the best among all tested models) identified models 1–4 (Δ_i_<2) as the best supported by the data (Table 1). Model 1 was the most important in predicting the data, with an Akaike weight of 0.35, followed by model 2, with a weight of 0.21 (Table 1). The sum of Akaike weights (a measure of the relative importance of each independent variable in predicting the data) identified the density of rural houses, NDVI, elevation and the percentage of land covered by crops (all with Σ ωi(j)>0.95) as the most important in explaining the prevalence of domestic infestation by *T. infestans* (Table 1). Model 1 had a pseudo-R^2^ of 0.19. When the model predictions were compared with the test dataset, a significant deviation between model and data was observed at high (>60%) infestation values (Figure S3), indicating a poor fit to the data. The occurrence of many infested communities with very few (1–3) houses and high infestation prevalence (but low overall contribution to the pattern of *T. infestans* distribution) explains the model's poor fit at high infestation levels.

**Table 1:** Factors associated with the high prevalence of domestic infestation by *T. infestans* in the Moreno Department, Santiago del Estero, Argentina.

	Variables analyzed^1^									Model fit		
Model	Den	Dist	LST	NDVI	Elev	Deg	Def	Crops	Constant	*F*	Δ_i_ 2	ω_i_
1	+X**	—	—	+X*	−X**	+X^NS^	+X*	−X**	+X*	6.25**	0	0.355
2	+X**	—	+X^NS^	+X**	−X**	—	—	−X**	−X	6.9**	1.1	0.205
3	+X**	−X^NS^	+X^NS^	+X*	−X*	+X^NS^	+X^NS^	−X*	−X	5.0**	1.5	0.168
4	+X**	—	+X^NS^	+X**	−X**	+X*	+X*	−X*	−X	5.0**	2	0.131
5	+X**	—	+X^NS^	+X*	−X**	+X	—	−X*	−X	5.8**	2.8	0.087
6	+X*	—	+X^NS^	—	−X**	+X**	+X**	−X*	−X	5.1**	6.2	0.016
7	—	—	+X^NS^	+X*	−X**	+X**	+X**	−X*	−X	4.8**	7.5	0.008
8	+X**	—	+X^NS^	+X*	—	+X^NS^	+X^NS^	−X^NS^	−X**	4.4**	5.4	0.024
9	+X**	—	+X^NS^	+X^NS^	−X**	+X**	+X^NS^	—	−X*	4.7**	8.3	0.006
10	—	—	+X^NS^	+X*	−X**	—	+X^NS^	−X*	−X**	4.5**	11.4	0.001
Σ ω_i_(*j*)	0.990	0.168	0.645	0.984	0.976	0.794	0.708	0.994	1.00			

1 Variables: Den, density of rural houses (# per sq. km); Dist, distance from a community to the nearest T. infestans infested community (meters); LST, mean maximum land surface temperature (°C); NDVI, Normalized Difference Vegetation Index (no units); Elev, mean elevation of each community (meters above sea level); Deg, percentage of landscape within 2 km of a village that was degraded (see text for details); Def, percentage of landscape within 2 km of a community that was deforested (see text for details); Crops, percentage of landscape within 2 km of a village that was modified for soy production.

Symbols: X (variable tested in model); — (variable not tested in model); − (negative association) + (positive association);

** (*P*≤0,01);

* (0,01<*P*≤0,05); NS (not significant).

Δ_i_ = AIC_i_−AIC_min_.

ω_i_ = exp (−1/2 Δ_i_)/Σ exp (−1/2 Δ_i_).

Σ ω_i_(*j*): sum of ω_i_ values from every model in which variable *i* was present. Indicates the relative importance of each independent variable in predicting the data.

2 Lowest AIC = 701.8.

The Akaike weight identified model 1 (ω_i_ = 0.68) as the best logistic model predicting the membership of a community in a cluster of high domestic infestation (Table 2). Elevation and percentage of landscape modified for soy production (negatively) and density of rural houses, mean maximum LST, NDVI, percentage of degraded and deforested lands (positively) were significantly associated with the membership of a community in a cluster of high *T. infestans* domestic infestation. The sum of Akaike weights identified distance to the nearest infested community as the least important factor in explaining membership in a cluster (Σw*_i_*(*j*) = 0.25; Table 1). The remaining variables all had Akaike weights higher than 0.9, indicating their strong influence in predicting the data. Model 1 (Table 3) classified correctly 98.4% of the communities in the training dataset, had a sensitivity of 93.3%, a specificity of 95.4% and a pseudo-R^2^ value of 0.72. Based on the model's sensitivity and specificity, the Youden index identified predicted probabilities higher than 0.404 as belonging to a cluster of high domestic infestation. Based on such criteria, a total of 88.6% of the 44 communities selected as test dataset were correctly classified by the model (Table S1). The model's sensitivity and specificity in predicting the training data were 0.86 and 0.9, respectively (Table S1).

**Table 2:** Factors associated with membership of a community in a cluster of high *T. infestans* infestation in the Moreno Department, Santiago del Estero, Argentina.

	Variables analyzed^1^									Model fit		
Model	Den	Dist	LST	NDVI	Elev	Deg	Def	Crops	Constant	?^2^	Δ_i_ 2	ω_i_
1	+X**	—	+X**	+X**	−X**	+X*	+X*	−X*	−X	154**	0.0	0.68
2	+X**	−X^NS^	+X**	+X**	−X**	+X*	+X*	−X**	−X	154**	2.1	0.25
3	+X**	—	+X**	+X**	−X**	—	—	−X*	−X	144**	6.1	0.03
4	+X**	—	+X**	+X*	−X**	+X	—	−X*	−X	146**	6.3	0.03
6	+X**	—	—	+X**	−X**	+X**	+X**	−X**	+X**	143**	9.1	0.01
5	+X**	—	+X*	—	−X**	+X**	+X**	−X*	−X	131**	20.1	2.90E-05
7	+X**	—	+X*	+X*	−X**	+X**	+X*	—	−X*	123**	28.9	3.60E-07
8	—	—	+X**	+X*	−X**	+X**	+X**	−X*	−X*	106**	46.0	1.00E-10
9	—	—	+X**	+X*	−X**	—	+X^NS^	−X*	−X**	82**	67.2	2.60E-15
10	+X**	—	+X**	+X*	—	+X^NS^	+X^NS^	−X^NS^	−X**	97**	55.1	7.30E-13
Σ ω_i_(*j*)	1.00	0.25	0.99	0.99	0.99	0.94	0.96	0.99				

1 Variables: Den, density of rural houses (# per sq. km); Dist, distance from a community to the nearest T. infestans infested community (meters); LST, mean maximum land surface temperature (°C); NDVI, Normalized Difference Vegetation Index (no units); Elev, mean elevation of each community (meters above sea level); Deg, percentage of landscape within 2 km of a village that was degraded (see text for details); Def, percentage of landscape within 2 km of a community that was deforested (see text for details); Crops, percentage of landscape within 2 km of a village that was modified for soy production.

Symbols: X (variable tested in model); — (variable not tested in model); − (negative association) + (positive association);

** (*P*≤0,01);

* (0,01<*P*≤0,05); NS (not significant).

Δ_i_ = AIC_i_−AIC_min_.

ω_i_ = exp (−1/2 Δ_i_)/Σ exp (−1/2 Δ_i_).

Σ ω_i_(*j*): sum of ω_i_ values from every model in which variable *i* was present. Indicates the relative importance of each independent variable in predicting the data.

2 Lowest AIC = 60.8.

**Table 3:** Best fitting multiple logistic regression model predicting membership in a cluster of high *T. infestans* domestic infestation.

					95% confidence interval	
Variable^1^	Coefficient	S.E.	*z*	*P*	Low	High
Dens	40.40	11.10	3.63	<0.001	18.54	62.19
Elev	−0.37	0.07	−4.34	<0.001	−0.42	−0.16
LST	0.91	0.33	2.78	0.005	0.27	1.55
NDVI	59.76	14.96	3.99	<0.001	30.43	89.08
Crops	−1.48	0.57	−2.57	0.001	−2.60	−0.35
Deg	0.12	0.05	2.50	0.012	0.03	0.22
Def	0.13	0.05	2.43	0.015	0.03	0.23
Constant	−7.40	12.01	−0.62	0.538	−30.94	16.13

1 Variables: Den, density of rural houses (# per sq. km); LST, mean maximum land surface temperature (°C); NDVI, Normalized Difference Vegetation Index (no units); Elev, mean elevation of each community (meters above sea level); Deg, percentage of landscape within 2 km of a village that was degraded (see text for details); Def, percentage of landscape within 2 km of a community that was deforested (see text for details); Crops, percentage of landscape within 2 km of a village that was modified for soy production.

### Risk maps of domestic infestation

**Figure 3:**
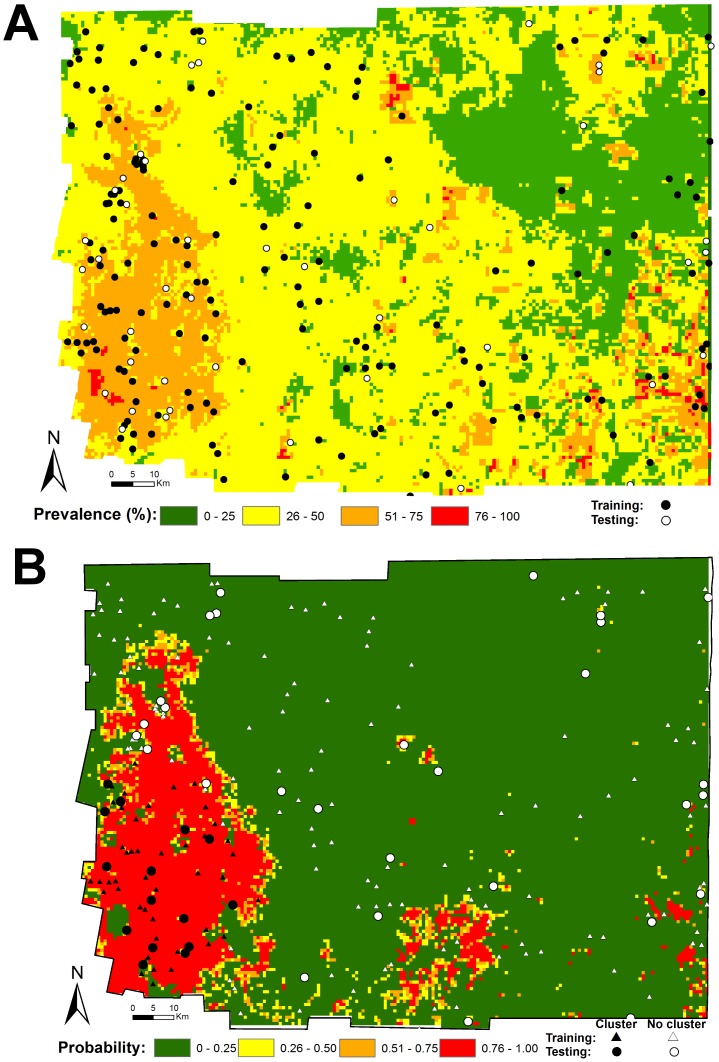
Risk maps of *T. infestans* domestic infestation. (A) Map showing the predicted prevalence of domestic infestation. (B) Map showing the probability of membership in a cluster of high domestic infestation. Both maps were estimated from the coefficients of the best fitting models. The spatial resolution of the map is 1×1 km.

The regression coefficients of the best models and their respective rasterized GIS layers were used to generate maps describing the predicted prevalence of *T. infestans* domestic infestation (Figure 3A) and the probability of membership in a cluster of *T. infestans* infestation (Figure 3B). Both maps outline the importance of environmental and demographic factors in defining the western extreme of the Department as a hot-spot (high-risk area) of bug infestation. Based on Figure 3B we estimated the high-risk area (probabilities >0.404, based on Youden index) to encompass approximately 2,736 km^2^, or 16% of the 16,788 km^2^ comprising the Moreno Department.

**Figure 4:**
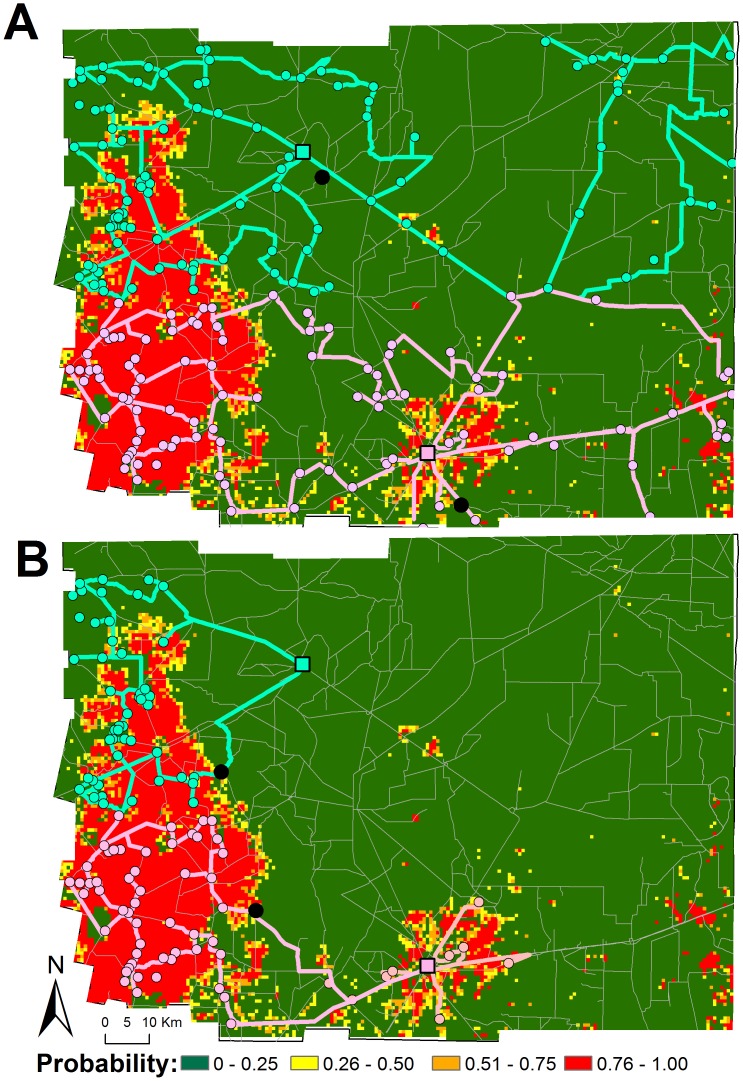
Spatially explicit insecticide spraying schemes in the Moreno Department. (A) Implementation of a spatially contiguous strategy (i.e., visiting the nearest neighbor of each community). (B) Strategy targeting interventions according to risk (i.e., only high-risk communities are treated). Color squares indicate the location of Moreno's main cities (Quimili in pink and Tintina in light blue) where spraying teams initiate their journeys. Spraying was performed by two trucks (one stationed on each city) with two technicians each (represented by lines of the same color as the square indicating the city where they are based at). Black circles indicate the communities first visited by each spraying team in each control scenario.

We used the risk map to assess the operational costs of reaching high-risk communities by simulating two vector control scenarios (Figure 4 and Table 4). When a blanket insecticide campaign was simulated, the first communities to be reached (black dots in Figure 4A) were located in close proximity to the two cities, in areas of predicted low probability of *T. infestans* domestic infestation clustering. From such communities, the spraying teams continued moving towards locations of no domestic infestation clustering located in the east (Figure 4A). It took the contiguous strategy 373 days and 2,063 km to reach and spray all communities (Table 4). The spatially targeted strategy, however, lasted for 190 days and covered 846 km (Table 4). More importantly, our analysis shows that ∼80% of all communities and households slated for spraying could be reached when targeting the areas predicted as high risk of domestic infestation clustering (representing only ∼16% of the Department's surface) (Figure 4B). Focusing efforts on the high-risk areas did not increase the average costs of treating each community (US$317 for blanket and US$312 for targeted interventions) or household (US$26.4 for blanket and US$25.9 for targeted interventions), but the reduced area and number of communities that need to be covered provides both a logistically and economically feasible alternative to blanket insecticide spraying, given limited resources.

**Table 4:** Assessing the costs of spraying communities predicted to be at high-risk of domestic infestation clustering.

Modeled scenario^1^	Location^2^	Total number of sprayed communities (houses)	Spraying coverage of all communities requiring blanket spraying^3^	Spraying coverage of all houses requiring spraying^4^	Distance covered (km)	Campaign duration (workdays)
Blanket^5^						
	Tintina	108 (1,391)	100	100	1,048	373
	Quimili	112 (1,244)	100	100	1,015	332
	All	**220 (2,635)**	**100**	**100**	**2,063**	**373**
Targeted^5^						
	Tintina	52 (584)	78.8	66.4	301	157
	Quimili	62 (789)	93.9	95.9	545	190
	All	**114 (1,373)**	**80.3**	**80.7**	**846**	**190**

1 Assumes all communities are visited. Blanket control is performed based on the rule of contiguity (i.e. the nearest neighbor first). Targeted control assumes only communities predicted as high-risk (from the risk map) are visited.

2 Refers to the city where spraying brigades are based.

3 Communities with prevalence of domestic infestation by *T. infestans* higher than 10% are slated for blanket spraying (Tintina = 66 communities and 880 houses; Quimili = 76 communities and 822 houses).

4 Selected from communities estimated in 3.

5 The total cost for a Blanket contiguous strategy was estimated to be US$69,779 and for a Targeted strategy US$35,552. Costs were based on Vazquez-Prokopec et al. 2009 [5] estimates and include cost of insecticides (US$6.9 per sprayed house), salaries (US$22 per-diem and US$11.2 wages per technician per day) and mobility (US$1 per km).

## Discussion

To achieve significant levels of control in highly endemic areas, Chagas disease vector control actions need to be sustained over time [3], [4], [38]. In the Gran Chaco, the current scenario of partial decentralization of health services, chronic rural poverty, lack of political support and very limited funding for vector control result in a serious challenge for the sustainable control of *T. infestans* and Chagas disease. The implementation of strategies that aim to obtain the greatest long-term impact with the scarce resources available represents a key challenge (and operational mandate) for Chagas disease vector control programs in the Gran Chaco and elsewhere. In the present study we show that domestic infestation with *T. infestans* is spatially heterogeneous at the district level, and that such heterogeneity can be taken into account to generate risk maps of domestic infestation that can help delineate spatially-targeted interventions.

In the southern extreme of the Gran Chaco (semiarid sub-region), the spatial distribution of *T. infestans* was found to be highly heterogeneous at the district level [7], [18]. Highly infested communities formed clusters ranging in extent from 4.5 to 57.7 km (median = 23.9 km; Q1–Q3 = 12.1–39.3 km) [7], [18]. Similarly, clustering of domestic infestation in Moreno was highest up to a distance of 24 km. These findings, obtained from two distant areas, suggest that spatial heterogeneity may be a common trait of *T. infestans* distribution at the district level and that, by identifying the conditions that favor bug infestation clustering, the entomologic risk for Chagas disease can be inferred at an operationally adequate spatial scale. As transmission of *T. cruzi* in the Gran Chaco occurs primarily inside rural domiciles and is highly correlated with *T. infestans* abundance [4],[39], mapping domestic infestation clustering in endemic areas constitutes a strong surrogate of Chagas disease transmission risk. If extrapolated to other areas within the Dry Chaco and carefully field-validated, the developed risk maps could be used as an operational tool for mapping bug infestation and parasite transmission risk and structuring vector control and disease surveillance.

Temperature and NDVI were two factors strongly associated with *T. infestans* spatial distribution in Moreno, as observed elsewhere [7], [40]. In Moreno, domestic infestation clustering was additionally associated to low elevations and degraded landscapes. In the Chaco region, elevation determines drainage and land productivity; elevated lands tend to be more productive for soy and cotton production than low –flood-prone– lands [29]. The expansion of the agricultural frontier –primarily transgenic soy– into eastern and central Moreno (where elevation and rainfall tend to be higher than in the west) during the past 15 years has brought a significant change in land tenure, landcover structure and socio-economic status of local populations. Fencing of large land parcels was followed by the displacement of local communities within them, the replacement of mud and thatch huts by better built houses to accommodate farm owners and employees, and the complete deforestation (often by slash and burn) of fenced areas for soybean production. Conversely, rural villagers living in the western extreme of the Moreno Department –too dry and flood-prone for soy production– had historically relied on a subsistence economy based mostly on extensive goat breeding, charcoal and fuel wood production, and hunting and gathering [3]. The low income and productivity associated with such activities translated into structural poverty which was exacerbated by the overexploitation of natural resources around rural communities [3]. Elucidating the intricate connections between environmental and social components of health [41] may allow a better understanding of *T. cruzi* transmission dynamics in the Gran Chaco.

Most of the communities for which no infestation data were available were isolated households or small groups of households (with up to 4 houses) located in the eastern extreme of Moreno. Although excluding such locations could have biased our analyses, we consider the results of our spatial analysis and logistic regression models are strong enough to conclude they were located near communities predicted to have very low or null *T. infestans* infestation. As new data for Moreno become available, we may refine our models to assess the infestation values in such “missing” communities. Another limitation to our study has been the reliance on householders' reports to assess *T. infestans* domestic infestation. Householders' collections in domiciles, although as sensitive as timed-manual collections at moderate or high bug infestations levels [31], lack sensitivity when bug abundance inside houses is very low or when it occurs in very cluttered rooms. Given the lack of evidence supporting that householders' sensitivity is community-dependent we conclude that, although our results likely underestimate the real domestic infestation prevalence, they reflect the spatial variation in *T. infestans* infestation levels across the Department.

### Vector control implications of spatially targeted interventions

In areas of low-to-moderate Chagas endemicity, where triatomine bug infestations tend to aggregate in certain households, communities or districts, targeting surveillance and control interventions in areas predicted at high risk can increase the coverage and cost-effectivenes of interventions [42], [43]. A recent study has found that targeted control of *Triatoma dimidiata* could be significantly improved when environmental risk factors (instead of the sole consideration of household factors such as building materials) are incorporated into proper statistical models [43]. Under this low-transmission context, the success and sustainability of targeted approaches thus relies on thorough epidemiological investigations, proper data collection and analysis and constant assessment of potential changes in the eco-epidemiological dimensions of vector distribution and parasite transmission [42], [43].

**Figure 5:**
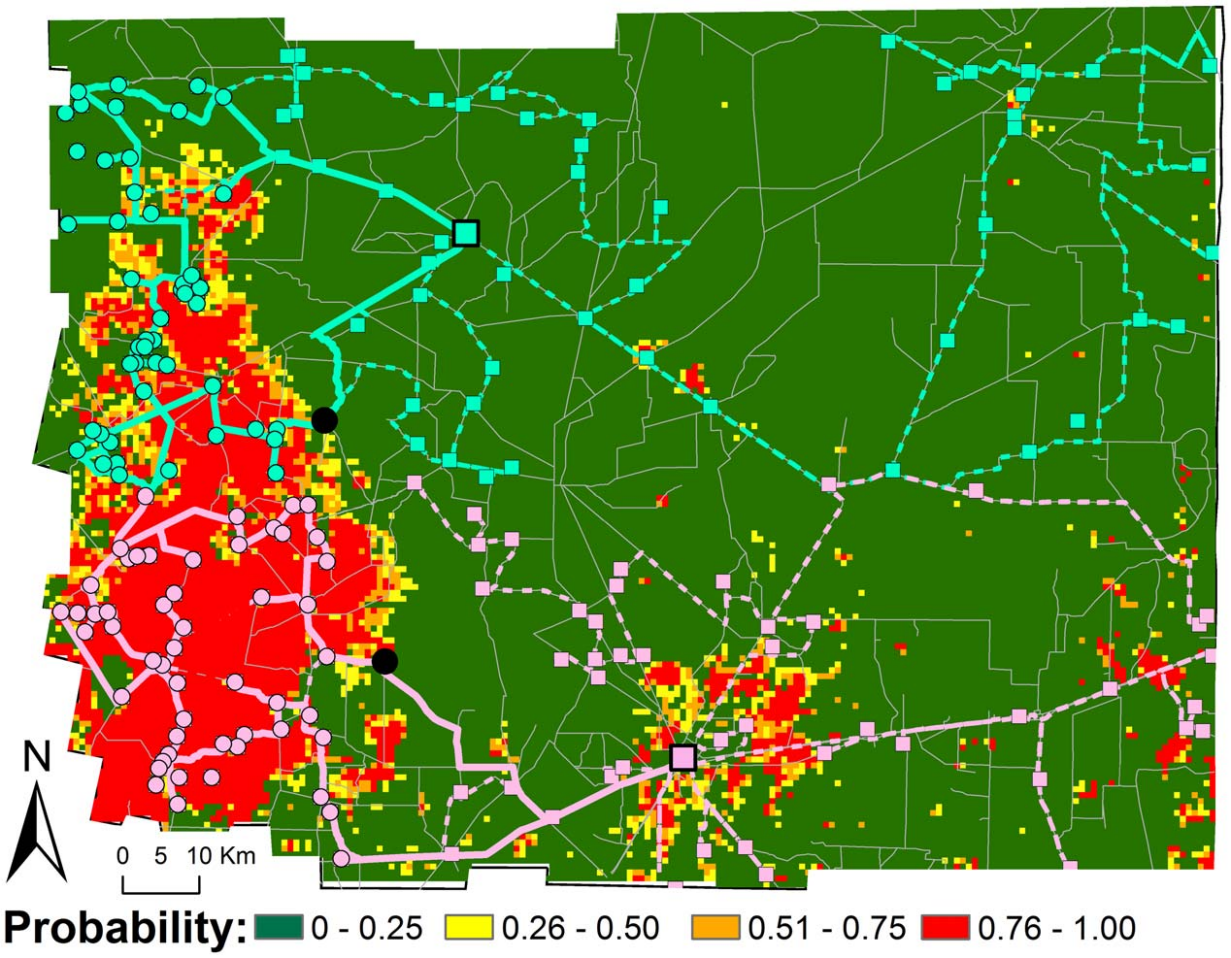
A spatially structured mixed vector control strategy. The proposed mixed strategy involves vertical control targeted at areas of predicted high risk of domestic infestation clustering (circles and solid lines) and horizontal control based on community participation in the communities predicted to be at medium to low risk (squares and dashed lines).

In highly endemic areas such as the Moreno Department and most of the Dry Chaco a true conflict between the need for high coverage of interventions and limited availability of resources exists. Most vertically structured control programs thus fail to reach all communities (or districts) at most risk. As an example, Santiago del Estero Province has recently invested significant resources to achieve full insecticide coverage but, given logistic limitations, excluded Moreno and other highly endemic Departments from the current priority areas. Targeting areas of presumed high risk alone while leaving others off the control scheme is not a feasible option. With the limited personnel and resources available, targeting vertical residual insecticide applications on Moreno communities predicted at high risk (∼80% of all infested communities slated for full spraying) would have required half the time and personnel costs than a blanket strategy. Here, we propose that with the remaining resources and time, NCS can implement a community participatory approach [4], [5] in areas of predicted medium-low infestation risk (Figure 5). In Moreno, the implementation of a horizontal attack phase (i.e., where local villagers were trained in the use and in charge of the application of residual insecticides) showed significant impacts in reducing bug infestation and parasite transmission but lacked sustainability due to the limited oversight by NCS staff [5]. Implementing a mixed strategy as the one shown in Figure 5 may provide a reasonable tradeoff between campaign costs, insecticide coverage and NCS staff involvement in control activities.

Targeted interventions are not a panacea. Relying on model predictions to infer where high risk areas occur may leave many infested communities excluded from the control scheme (as observed in central Moreno). Missing or excluding communities could increase their risk of parasite transmission (due to relaxation from insecticide pressure) and contribute to community discomfort due to the perception that control programs do not care about them. Furthermore, bug reinfestation in communities located in the border of high risk areas may proceed faster due to a high likelihood of *T. infestans* flight or passive dispersal from untreated communities nearby [26]. Transportation models like the one developed in this study can help bridge some of these challenges only when they are based on well developed predictive models and accurate road network information. Only after thorough field validation spatially targeted approaches may be considered as a feasible operational alternative to blanket vertical control in districts like Moreno.

Attaining the sustainable control of *T. infestans* and management of Chagas disease in highly endemic areas such as the economically deprived Gran Chaco not only will require the consideration of biological features of *T. infestans* and socio-economic attributes of the local population unique to this region, but also the incorporation of novel methods and approaches to help vector control programs design rational and more cost-effective interventions.

## Supporting Information

Figure S1 **Distribution of communities and landscape characteristics of the Moreno Department, Santiago del Estero, Argentina.** Inset shows the location of Moreno within Santiago del Estero Province and Argentina.
(PDF)

Figure S2 **Maximum clustering distance.** Maximum absolute Gi*(d) values for the prevalence of *T. infestans* domestic infestation. The distance at which Gi*(d) reaches its maximum is considered the scale at which clustering of *T. infestans* prevalence is maximized. For Moreno, such distance was 24 km.
(PDF)

Figure S3 **Fit of the best model predicting domestic infestation prevalence.** Scatterplot showing the best multiple linear regression predicted prevalence of *T. infestans* domestic infestation versus the observed infestation in the test dataset. The model predictions were compared with a subset of 44 communities not included in the model (test dataset). The diagonal line indicates perfect agreement between model and data.
(PDF)

Table S1 **Classification matrix indicating the fit of the best logistic regression model predicting the membership in a cluster of high** ***T. infestans*** **domestic infestation.** The model predictions were compared with observed data from the test dataset (i.e., 44 communities not included in the model).
(DOCX)

## References

[1] Schofield CJ, Jannin J, Salvatella R (2006) The future of Chagas disease control. Trends Parasitol 22: 583–588. 17049308 10.1016/j.pt.2006.09.011

[2] World Health Organization (2002) Control of Chagas disease: second report of the WHO expert committee. Geneva: World Health Organization. vi, 109 p.

[3] Gürtler RE (2009) Sustainability of vector control strategies in the Gran Chaco Region: current challenges and possible approaches. Mem Inst Oswaldo Cruz 104 Suppl 1: 52–59. 19753458 10.1590/s0074-02762009000900009 PMC3072747

[4] Gürtler RE, Kitron U, Cecere MC, Segura EL, Cohen JE (2007) Sustainable vector control and management of Chagas disease in the Gran Chaco, Argentina. Proc Natl Acad Sci USA 104: 16194–16199. 17913895 10.1073/pnas.0700863104 PMC2042184

[5] Vazquez-Prokopec GM, Spillmann C, Zaidenberg M, Kitron U, Gürtler RE (2009) Cost-effectiveness of chagas disease vector control strategies in Northwestern Argentina. PLoS Negl Trop Dis 3: e363. 19156190 10.1371/journal.pntd.0000363 PMC2613538

[6] Cecere MC, Vazquez-Prokopec GM, Ceballos LA, Gurevitz JM, Zarate JE, et al (2006) Comparative trial of effectiveness of pyrethroid insecticides against peridomestic populations of *Triatoma infestans* in northwestern Argentina. J Med Entomol 43: 902–909. 17017227 10.1603/0022-2585(2006)43[902:ctoeop]2.0.co;2 PMC1894891

[7] Porcasi X, Catala SS, Hrellac H, Scavuzzo MC, Gorla DE (2006) Infestation of rural houses by *Triatoma infestans* (Hemiptera: Reduviidae) in southern area of Gran Chaco in Argentina. J Med Entomol 43: 1060–1067. 17017246 10.1603/0022-2585(2006)43[1060:iorhbt]2.0.co;2

[8] Gürtler RE, Canale DM, Spillmann C, Stariolo R, Salomon OD, et al (2004) Effectiveness of residual spraying of peridomestic ecotopes with deltamethrin and permethrin on *Triatoma infestans* in rural western Argentina: a district-wide randomized trial. Bull World Health Organ 82: 196–205. 15112008 PMC2585938

[9] Ceballos LA, Piccinali RV, Berkunsky I, Kitron U, Gürtler RE (2009) First finding of melanic sylvatic *Triatoma infestans* (Hemiptera: Reduviidae) colonies in the Argentine Chaco. J Med Entomol 46: 1195–1202. 19769054 10.1603/033.046.0530 PMC2782367

[10] Noireau F, Flores R, Gutierrez T, Abad-Franch F, Flores E, et al (2000) Natural ecotopes of *Triatoma infestans* dark morph and other sylvatic triatomines in the Bolivian Chaco. Trans Roy Soc Trop Med Hyg 94: 23–27. 10748892 10.1016/s0035-9203(00)90426-7

[11] Rolon M, Vega MC, Roman F, Gomez A, Rojas de Arias A (2011) First report of colonies of sylvatic *Triatoma infestans* (Hemiptera: Reduviidae) in the Paraguayan Chaco, using a trained dog. PLoS Negl Trop Dis 5: e1026. 21572522 10.1371/journal.pntd.0001026 PMC3086807

[12] Germano MD, Roca Acevedo G, Mougabure Cueto GA, Toloza AC, Vassena CV, et al (2010) New findings of insecticide resistance in *Triatoma infestans* (Heteroptera: Reduviidae) from the Gran Chaco. J Med Entomol 47: 1077–1081. 21175056 10.1603/me10069

[13] Lardeux F, Depickere S, Duchon S, Chavez T (2010) Insecticide resistance of *Triatoma infestans* (Hemiptera, Reduviidae) vector of Chagas disease in Bolivia. Trop Med Int Health 9: 1037–1048. 10.1111/j.1365-3156.2010.02573.x 20545921

[14] Picollo MI, Vassena C, Orihuela PS, Barrios S, Zaidemberg M, et al (2005) High resistance to pyrethroid insecticides associated with ineffective field treatments in *Triatoma infestans* (Hemiptera: Reduviidae) from Northern Argentina. J Med Entomol 42: 637–642. 16119553 10.1093/jmedent/42.4.637

[15] Vassena C, Picollo M, Zerba E (2000) Insecticide resistance in Brazilian *Triatoma infestans* and Venezuelan *Rhodnius prolixus* . Med Vet Entomol 14: 51–55. 10759312 10.1046/j.1365-2915.2000.00203.x

[16] Ceballos LA, Vazquez-Prokopec GM, Cecere MC, Marcet PL, Gürtler RE (2005) Feeding rates, nutritional status and flight dispersal potential of peridomestic populations of *Triatoma infestans* in rural northwestern Argentina. Acta Trop 95: 149–159. 15993834 10.1016/j.actatropica.2005.05.010

[17] Cardinal MV, Castanera MB, Lauricella MA, Cecere MC, Ceballos LA, et al (2006) A prospective study of the effects of sustained vector surveillance following community-wide insecticide application on *Trypanosoma cruzi* infection of dogs and cats in rural Northwestern Argentina. Am J Trop Med Hyg 75: 753–761. 17038707 PMC1853286

[18] Gorla DE, Porcasi X, Hrellac H, Catala SS (2009) Spatial stratification of house infestation by *Triatoma infestans* in La Rioja, Argentina. Am J Trop Med Hyg 80: 405–409. 19270290

[19] Porcasi X, Hrellac H, Catalá S, Moreno M, Abrahan L, et al (2007) Infestation of rural houses by *Triatoma infestans* in the region of Los Llanos (La Rioja, Argentina). Mem Inst Oswaldo Cruz 102: 63–68. 17294001 10.1590/s0074-02762007000100010

[20] Cecere MC, Vazquez-Prokopec GM, Gürtler RE, Kitron U (2004) Spatio-temporal analysis of reinfestation by Triatoma infestans (Hemiptera: Reduviidae) following insecticide spraying in a rural community in northwestern Argentina. Am J Trop Med Hyg 71: 803–810. 15642975 PMC1351234

[21] Levy MZ, Malaga Chavez FS, Cornejo Del Carpio JG, Vilhena DA, McKenzie FE, et al (2010) Rational spatio-temporal strategies for controlling a Chagas disease vector in urban environments. J Roy Soc Interface 7: 1061–1070. 20061346 10.1098/rsif.2009.0479 PMC2880077

[22] Barbu C, Dumonteil E, Gourbiere S (2011) Evaluation of spatially targeted strategies to control non-domiciliated *Triatoma dimidiata* vector of Chagas disease. PLoS Negl Trop Dis 5: e1045. 21610862 10.1371/journal.pntd.0001045 PMC3096612

[23] Cecere M, Gürtler R, Canale D, Chuit R, Cohen J (2002) Effects of partial housing improvement and insecticide spraying on the reinfestation dynamics of *Triatoma infestans* in rural northwestern Argentina. Acta Trop 1–16. 10.1016/s0001-706x(02)00183-3 12429427

[24] Gürtler RE, Cecere MC, Rubel DN, Schweigmann NJ (1992) Determinants of the domiciliary density of *Triatoma infestans*, vector of Chagas disease. Med Vet Entomol 6: 75–83. 1600232 10.1111/j.1365-2915.1992.tb00039.x

[25] Gürtler RE, Chuit R, Cecere MC, Castañera MB, Cohen JE, et al (1998) Household prevalence of seropositivity for *Trypanosoma cruzi* in three rural villages in northewestern Argentina: environmental demographic, and entomologic associations. Am J Trop Med Hyg 59: 741–749. 9840591 10.4269/ajtmh.1998.59.741

[26] Cecere MC, Vasquez-Prokopec GM, Gürtler RE, Kitron U (2006) Reinfestation sources for Chagas disease vector, *Triatoma infestans*, Argentina. Emerg Infect Dis 12: 1096–1102. 16836826 10.3201/eid1207.051445 PMC1853288

[27] Levy M, Bowman N, Kawai V, Waller L, Cornejo del Carpio J, et al (2006) Periurban *Trypanosoma cruzi*-infected *Triatoma infestans*, Arequipa, Peru. Emerg Infect Dis 12: 1345–1352. 17073082 10.3201/eid1209.051662 PMC3294737

[28] Gürtler RE, Canale DM, Spillmann C, Stariolo R, Salomon OD, et al (2004) Effectiveness of residual spraying of peridomestic ecotopes with deltamethrin and permethrin on *Triatoma infestans* in rural western Argentina: a district-wide randomized trial. Bull World Health Organ 82: 196–205. 15112008 PMC2585938

[29] Bucher EH, Huszar PC (1999) Sustainable management of the Gran Chaco of South America: Ecological promise and economic constraints. J Environ Management 57: 99–108.

[30] Instituto Nacional de Estadisticas y Censos (2004) Anuario estadístico de la República Argentina.; INDEC. Buenos Aires: INDEC. 488 p.

[31] Gürtler RE, Cecere MC, Canale DM, Castañera MB, Chuit R, et al (1999) Monitoring house reinfestation by vectors of Chagas disease: a comparative trial of detection methods during a four-year follow-up. Acta Trop 72: 213–234. 10206120 10.1016/s0001-706x(98)00096-5

[32] Chavez P (1996) Image-Based Atmospheric Corrections-Revisited and Improved. PE&RS 62: 1025–1036.

[33] Waller LA, Gotway CA (2004) Applied spatial statistics for public health data: John Wiley & Sons.

[34] Ord JK, Getis A (1995) Local spatial autocorrelation statistics. Distributional issues and an application. Geogr Anal 27: 286–306.

[35] Burnham KP, Anderson DR (2002) Model selection and multimodel inference: a practical information-theoretic approach. New York: Springer. xxvi, 488 p.

[36] Fluss R, Faraggi D, Reiser B (2005) Estimation of the Youden Index and its associated cutoff point. Biom J 47: 458–472. 16161804 10.1002/bimj.200410135

[37] Little J, Murty K, Sweeney D, Karel C (1963) An Algorithm for the Traveling Salesman Problem. Operat Res 11: 972–989.

[38] Tarleton RL, Reithinger R, Urbina JA, Kitron U, Gurtler RE (2007) The challenges of Chagas Disease– grim outlook or glimmer of hope. PLoS Med 4: e332. 18162039 10.1371/journal.pmed.0040332 PMC2222930

[39] Gürtler RE, Cecere MC, Lauricella MA, Petersen RM, Chuit R, et al (2005) Incidence of *Trypanosoma cruzi* infection among children following domestic reinfestation after insecticide spraying in rural northwestern Argentina. Am J Trop Med Hyg 73: 95–103. 16014842 PMC1351233

[40] Gorla D (2002) Variables ambientales registradas por sensores remotos como indicadores de la distribución geográfica de *Triatoma infestans* (Heteroptera: Reduviidae). Ecología Austral 12: 117–127.

[41] Lebel J, Centre IDR (2003) Health: an ecosystem approach: International Development Research Centre.

[42] Nakagawa J, Hashimoto K, Cordon-Rosales C, Abraham Juarez J, Trampe R, et al (2003) The impact of vector control on *Triatoma dimidiata* in the Guatemalan department of Jutiapa. Ann Trop Med Parasitol 97: 288–297. 12803860 10.1179/000349803235001895

[43] King R, Cordon-Rosales C, Cox J, Davies CR, Kitron U (2011) *Triatoma dimidiata* infestation in Chagas disease endemic regions of Guatemala: comparison of random and targeted cross-sectional surveys. PLoS Negl Trop Dis 5: e1035. 21532742 10.1371/journal.pntd.0001035 PMC3075228

