# Supplementary material for: Spatial Heterogeneity and Risk Maps of Community Infestation by Triatoma infestans in Rural Northwestern Argentina
Source: PLoS Negl Trop Dis. 2012 Aug 14;6(8):e1788. doi: 10.1371/journal.pntd.0001788 (PMC3419179; doi:10.1371/journal.pntd.0001788)

Figure S1. Distribution of communities and landscape characteristics of the Moreno Department, Santiago del Estero, Argentina. Inset shows the location of Moreno within Santiago del Estero Province and Argentina.

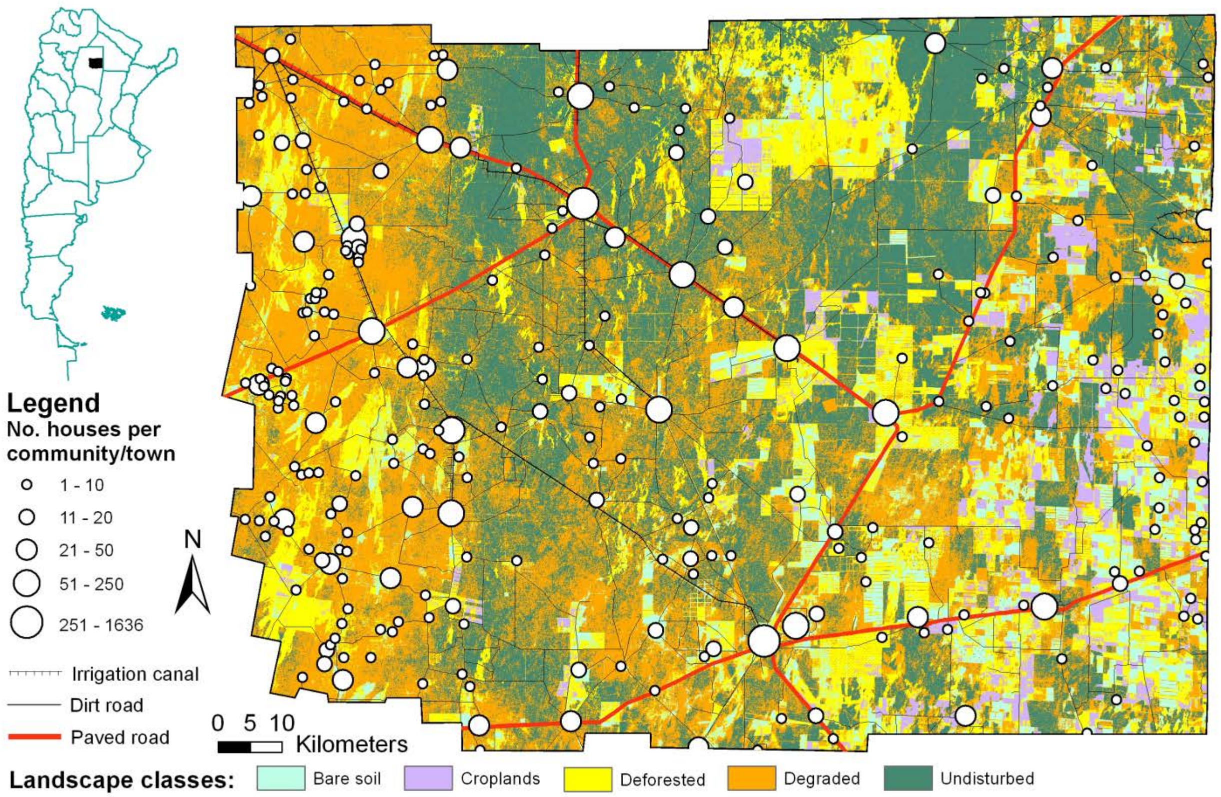

Supplement: Figure S1 — Distribution of communities and landscape characteristics of the Moreno Department, Santiago del Estero, Argentina. Inset shows the location of Moreno within Santiago del Estero Province and Argentina. (PDF) [file pntd.0001788.s001.pdf]
