# Supplementary material for: Spatial Heterogeneity and Risk Maps of Community Infestation by Triatoma infestans in Rural Northwestern Argentina
Source: PLoS Negl Trop Dis. 2012 Aug 14;6(8):e1788. doi: 10.1371/journal.pntd.0001788 (PMC3419179; doi:10.1371/journal.pntd.0001788)

Figure S2. Maximum absolute Gi*(d) values for the prevalence of *T. infestans* domestic infestation. The distance at which Gi*(d) reaches its maximum is considered the scale at which clustering of *T. infestans* prevalence is maximized. For Moreno, such distance was 24 km.

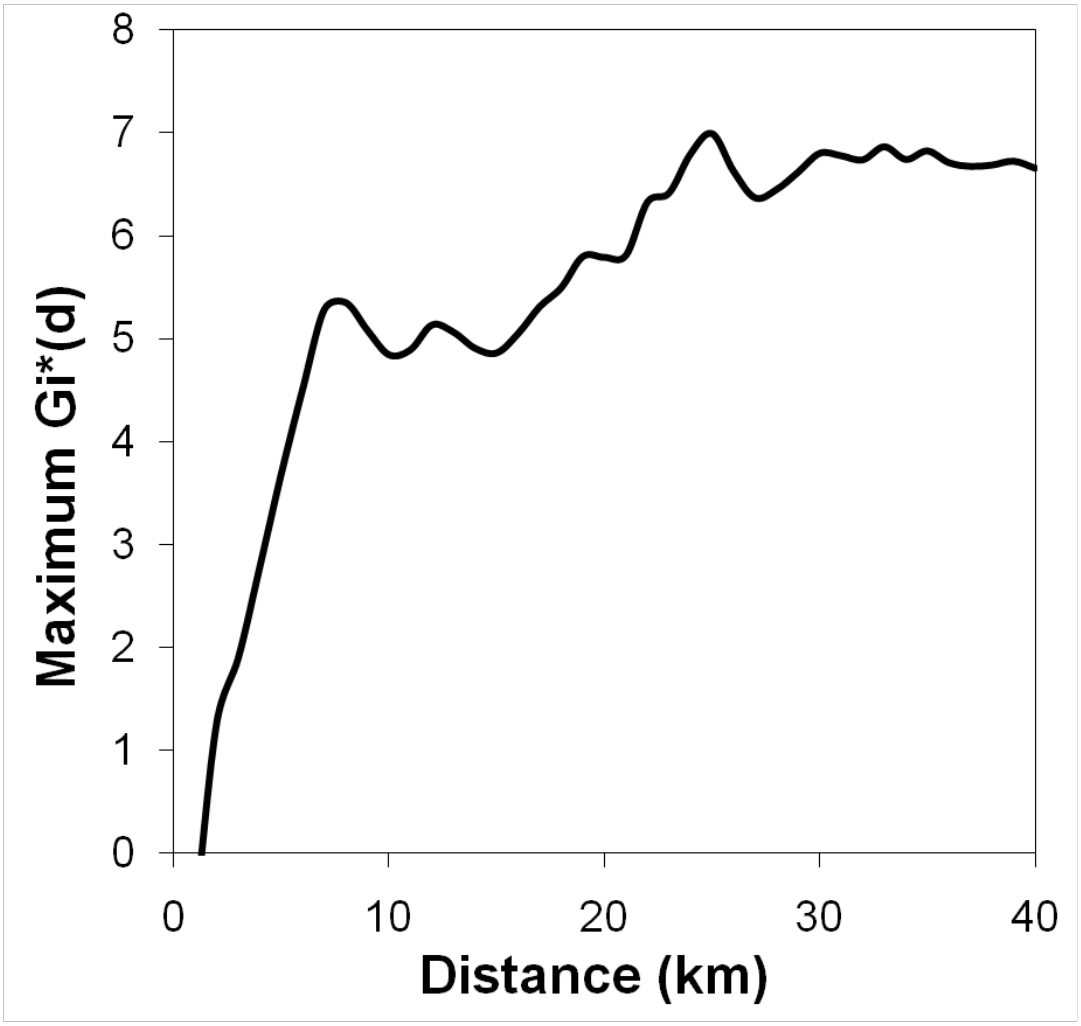

Supplement: Figure S2 — Maximum clustering distance. Maximum absolute Gi*(d) values for the prevalence of T. infestans domestic infestation. The distance at which Gi*(d) reaches its maximum is considered the scale at which clustering of T. infestans prevalence is maximized. For Moreno, such distance was 24 km. (PDF) [file pntd.0001788.s002.pdf]
