# Supplementary material for: Spatial Heterogeneity and Risk Maps of Community Infestation by Triatoma infestans in Rural Northwestern Argentina
Source: PLoS Negl Trop Dis. 2012 Aug 14;6(8):e1788. doi: 10.1371/journal.pntd.0001788 (PMC3419179; doi:10.1371/journal.pntd.0001788)

Figure S3. Scatterplot showing the best multiple linear regression predicted prevalence of *T. infestans* domestic infestation versus the observed infestation in the test dataset. The model predictions were compared with a subset of 44 communities not included in the model (test dataset). The diagonal line indicates perfect agreement between model and data.

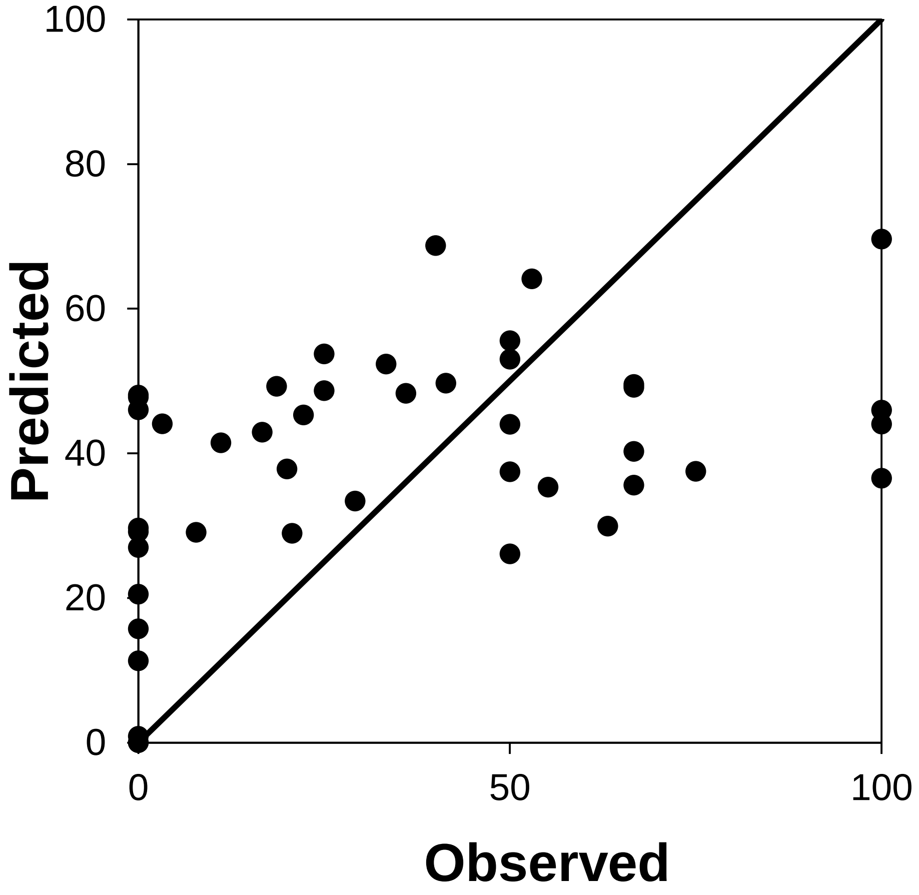

Supplement: Figure S3 — Fit of the best model predicting domestic infestation prevalence. Scatterplot showing the best multiple linear regression predicted prevalence of T. infestans domestic infestation versus the observed infestation in the test dataset. The model predictions were compared with a subset of 44 communities not included in the model (test dataset). The diagonal line indicates perfect agreement between model and data. (PDF) [file pntd.0001788.s003.pdf]
