# Supplementary material for: Spatial Heterogeneity and Risk Maps of Community Infestation by Triatoma infestans in Rural Northwestern Argentina
Source: PLoS Negl Trop Dis. 2012 Aug 14;6(8):e1788. doi: 10.1371/journal.pntd.0001788 (PMC3419179; doi:10.1371/journal.pntd.0001788)
Supplement: Table S1 — Classification matrix indicating the fit of the best logistic regression model predicting the membership in a cluster of high T. infestans domestic infestation. The model predictions were compared with observed data from the test dataset (i.e., 44 communities not included in the model). (DOCX) [file pntd.0001788.s004.docx]

**Table S1.** Classification matrix indicating the fit of the best logistic regression model predicting the membership in a cluster of high *T. infestans* domestic infestation. The model predictions were compared with observed data from the test dataset (i.e., 44 communities not included in the model).

|  |  | Predicted clustering | |  |
| --- | --- | --- | --- | --- |
|  |  | 1 | 0 | Total |
| Observed clustering | 1 | 12 | 3 | 15 |
|  | 0 | 2 | 27 | 29 |
| Total |  | 14 | 30 | 44 |
| _Sensitivity (95% CI), 0.857 (0.625-0.971); Specificity (95% CI): 0.900 (0.792-0.953); Kappa (95% CI): 0.743 (0.409-0.906). Chi2 = 25.354, df=1, P<0.001._ | | | | |
